# Changes in mental health and help-seeking among young Australian adults during the COVID-19 pandemic: a prospective cohort study

**DOI:** 10.1017/S0033291721001963

**Published:** 2021-05-10

**Authors:** Emily Upton, Philip J. Clare, Alexandra Aiken, Veronica C. Boland, Clara De Torres, Raimondo Bruno, Delyse Hutchinson, Kypros Kypri, Richard Mattick, Nyanda McBride, Amy Peacock

**Affiliations:** 1 National Drug and Alcohol Research Centre, University of New South Wales, Sydney, Australia; 2 Prevention Research Collaboration, The University of Sydney, Sydney, Australia; 3 School of Psychological Sciences, University of Tasmania, Tasmania, Australia; 4 Centre for Social and Early Emotional Development, Deakin University, Victoria, Australia; 5 Department of Paediatrics, The University of Melbourne, Parkville, Australia; 6 Murdoch Children's Research Institute, Royal Children's Hospital, The University of Melbourne, Parkville, Australia; 7 School of Medicine and Public Health, University of Newcastle, Callaghan, Australia; 8 National Drug Research Institute, Curtin University, Perth, Australia

**Keywords:** Anxiety, Australia, coronavirus infection, COVID-19, depression, mental health, prospective cohort, treatment, young adults

## Abstract

**Background:**

Young people may have elevated risk for poorer mental health during the coronavirus disease 2019 (COVID-19) pandemic, yet longitudinal studies documenting this impact are lacking. This study assessed changes in mental health and help-seeking since COVID-19 restrictions in young Australians, including gender differences.

**Methods:**

Data were drawn from a recent subsample (*n* = 443; 60% female; *M*_age_ = 22.0) of a prospective cohort originally recruited in secondary school to complete annual surveys. The subsample completed an additional COVID-19 survey during COVID-19 restrictions (May–June 2020), which was compared to responses from their latest annual survey (August 2019–March 2020). Mixed effect models with time and gender as the primary predictors were conducted for: (i) scores on the Patient Health Questionnaire Depression 9-item (PHQ-9) and Generalised Anxiety Disorder 7-item (GAD-7) modules assessed before and during COVID-19 restrictions, and (ii) self-reported help-seeking from a health professional in February 2020, and the month preceding May–June 2020.

**Results:**

Mean symptom scores increased from before to during COVID-19 restrictions on the PHQ-9 (coefficient: 1.29; 95% CI 0.72–1.86) and GAD-7 (0.78; 95% CI 0.26–1.31), but there was no increase in help-seeking over time (odds ratio 0.50; 95% CI 0.19–1.32). There was no evidence of differential changes by gender.

**Conclusions:**

This study found increases in depression and anxiety symptoms but not greater help-seeking among young Australian adults during the first wave of the pandemic. Increasing availability and awareness of accessible treatment options and psychoeducation is critical, as well as further research into risk and protective factors to help target treatment to this vulnerable age group.

## Background

There is growing evidence that the coronavirus disease 2019 (COVID-19) pandemic and associated social restrictions have had a negative impact on global mental health, with high levels of psychological distress seen in the general population since restrictions were implemented (e.g. Torales, O'Higgins, Castaldelli-Maia, & Ventriglio, 2020; Xiong *et al*. 2020). Poorer mental health may arise through various mechanisms, including fear of the disease (Ornell, Schuch, Sordi, & Kessler, 2020), increased social isolation (Clay & Parker, 2020; Pfefferbaum & North, 2020), impacts on employment and financial stress (Australian Bureau of Statistics (ABS), 2020; Power, Hughes, Cotter, and Cannon, 2020), and negative changes in health behaviours such as alcohol use (Furlong & Finnie, 2020; Stanton et al., 2020).

There is particular concern for the mental health of young people (aged 15–24 years; UNDESA, 2013) during the pandemic. The majority of mental health disorders emerge before 26 years of age (McGorry & Goldstone, 2011), and social isolation and loneliness were common among young people prior to the pandemic (Lim, Eres, & Peck, 2019). Young people have low engagement with mental health treatment (Gulliver, Griffiths, & Christensen, 2010; Wilson, 2010), and social distancing practices may be a further barrier (Power et al., 2020). Young people may also disproportionately experience certain stressors associated with the pandemic (Furlong & Finnie, 2020; Power et al., 2020). For example, industries most likely to report reduced staff working hours (e.g. hospitality) are those where the majority of young people are employed, often on a non-contract basis (ABS, 2020), increasing risk of financial stress. Young people are also likely to experience significant disruption to, or loss of, other structured activity such as tertiary education and workplace training (Power et al., 2020).

International cross-sectional studies show elevated depression, anxiety and stress amongst young adults during the pandemic (e.g. Alonzi, La Torre, & Silverstein, 2020; Liu, Zhang, Wong, & Hyun, 2020) but are limited by recall bias. A small but growing number of longitudinal studies show increased depression and anxiety symptomatology with the pandemic (Lee, Cadigan, & Rhew, 2020; Parola, Rossi, Tessitore, Troisi, & Mannarini, 2020; Shanahan et al., 2020). However, these studies have predominantly been conducted in North America and Western Europe: regions with high rates of COVID-19 cases and deaths (World Health Organisation (WHO), 2020). There is a lack of published longitudinal research from other countries such as Australia, which has had high testing and low rates of cases and deaths relative to the global average (WHO, 2020). This gap is critical to address to understand the mental health burden of the pandemic on younger people more broadly.

As well as younger age, gender has also been shown to be a key correlate of mental health outcomes during the pandemic, with women reporting higher levels of depression, anxiety and stress symptoms compared to men (ABS, 2020; Stanton et al., 2020; Xiong et al., 2020). These results align with research showing higher pre-existing prevalence of depression and anxiety in women than men (Leach, Christensen, Mackinnon, Windsor, & Butterworth, 2008; Piccinelli & Wilkinson, 2000), and reinforce the need to investigate longitudinally whether changes in mental health and help-seeking during the pandemic differ by gender.

We used data from the Australian Parental Supply of Alcohol Longitudinal Study (APSALS; Aiken et al., 2017) to investigate changes in mental health and help-seeking from before the pandemic (up to March 2020, the onset of the first wave of COVID-19 in Australia) to during the pandemic (May–June 2020, after implementation of national social restrictions in Australia). Depression and anxiety were targeted as key mental health outcomes given they are among the most common mental health disorders in young adults (McGorry & Goldstone, 2011; Wilson, Rickwood, Bushnell, Caputi, & Thomas, 2011). The aims were to assess: (1) changes in generalised anxiety and depression symptoms since COVID-19 restrictions, including both perceived and prospective changes; (2) changes in mental health help-seeking since COVID-19 restrictions, as well as engagement in self-help behaviours; and (3) changes in mental health and help-seeking since COVID-19 restrictions by gender.

## Method

### Sample

This study used the APSALS (registered at ClinicalTrials.gov: NCT02280551) cohort of 1927 young people (Aiken et al., 2017). Participants (current sample *M*_age_ = 22.0 years) and one parent/guardian were recruited via an opt-in process in 2010 and 2011 (*M*_age_ = 12.9 years) from grade 7 classes in Australian schools; the parent/guardian provided informed consent. Participants complete annual online or hardcopy surveys principally regarding alcohol use over the preceding 12 months; each annual follow-up survey is henceforth referred to by ‘Wave’ number (see online Supplementary Appendix B1 for study flowchart). Items on mental health were added in Wave 10 (commenced August 2019, data collection ongoing). Parents/guardians were only surveyed until Wave 5. Participants were reimbursed $AUD50 for Wave 10 ($AUD20 until Wave 8) and could win one of 10 $AUD500 gift vouchers.

When the first COVID-19 fatality was recorded in Australia (15 March 2020) and national social distancing restrictions had begun to be implemented^†^^1^, Wave 9 was complete (August 2018–December 2019) and Wave 10 was underway (commenced August 2019). Participants who had completed Wave 10 up to 14 March 2020 were invited to complete an additional 10-min online survey via Qualtrics assessing their experiences during COVID-19 restrictions (hereafter ‘COVID-19 survey’), with the opportunity to win a $AUD500 gift voucher. Of 813 participants invited, 443 completed COVID-19 surveys (54% response rate) between 18 May and 25 June 2020 (see online Supplementary Appendix A for figures showing how data collection related to the COVID-19 pandemic in Australia, and online Supplementary Appendix B for study flowchart). Data used here are drawn from Wave 10 and the COVID-19 survey mental health variables. Due to the wealth of data collected, data on changes in alcohol use and related harm with the COVID-19 pandemic and associated restrictions are considered in a separate paper (Clare et al., under review).

APSALS was approved by the University of New South Wales Human Research Ethics Committee and ratified by the universities of Tasmania, Newcastle, and Queensland, and Curtin University. Findings are reported according to the STROBE statement (online Supplementary Appendix B).

### Measures

Our analysis consisted of two outcome measures of generalised anxiety and depression assessed longitudinally, and various measures of mental health and help-seeking assessed in the COVID-19 survey only. Demographic and COVID-19-specific questions were asked in the COVID-19 survey; details of these measures are reported in online Supplementary Appendix C.

#### Measures of mental health

*Patient Health Questionnaire – Depression Module* (*PHQ-9*; Kroenke, Spitzer, and Williams, 2001). The PHQ-9 is a reliable and widely validated measure of depression symptoms (*α* = 0.81–0.87; Beard, Hsu, Rifkin, Busch, & Björgvinsson, 2016; Kroenke *et al*. 2001; Titov *et al*. 2011; current study *α* = 0.91). Its nine items are based on DSM-IV diagnostic criteria for major depressive disorder (American Psychiatric Association, 1994) and can be used to screen for presence of depression (cut-off score of ⩾ 10; Kroenke et al., 2001) and grade severity, with scores of 5, 10, 15 and 20 representing cut-off points for mild, moderate, moderately severe, and severe depression, respectively. Primary outcome variables were raw score (range: 0–27) and a binary indicator of meeting the screening cut-off score of 10; a categorical variable corresponding to severity categories (none, mild, etc.) was used for sensitivity analyses.

*Generalised Anxiety Disorder 7-item* (*GAD-7*; Spitzer, Kroenke, Williams, and Löwe, 2006). The GAD-7 is a brief self-report scale with strong reliability and validity in identifying probable DSM-IV generalised anxiety disorder (GAD; American Psychiatric Association, 1994), as well as indicating severity, in both psychiatric and general populations (*α* = 0.89–0.92; Löwe *et al*. 2008; Plummer, Manea, Trepel, & McMillan, 2016; Spitzer *et al*. 2006; current study *α* = 0.93). Scores of 5, 10, and 15 reflect mild, moderate, or severe levels of symptomatology, respectively, and scores of ⩾10 indicate probable GAD (Löwe et al., 2008; Spitzer et al., 2006). Primary outcome variables were raw score (range: 0–21) and a binary indicator of meeting the screening cut-off score of 10; a categorical variable corresponding to severity categories (mild, moderate, severe) was used for sensitivity analyses.

*Self-rated mental health.* Participants were asked to self-rate their current mental health using a single-item measure commonly used in population health research (Ahmad, Jhajj, Stewart, Burghardt, & Bierman, 2014), on a five-point Likert scale from *Excellent* to *Poor*. This single-item measure has good validity and correlates well with other standardised measures of mental health (Ahmad et al., 2014). They were separately asked whether they believed their mental health had changed due to the COVID-19 crisis (*Much Worse, Somewhat Worse, Same, Somewhat Better, Much Better*). Responses were aggregated into three categories (*Worse, Same, Better*) for analysis. These items were included in the COVID-19 survey only.

#### Measures of help-seeking and self-help

*Help-seeking.* Participants were asked whether they had sought help for their mental health from a general practitioner or other qualified health professional (e.g. psychologist) in February and the month prior to completing the COVID-19 survey.

*Self-help behaviours.* In the COVID-19 survey, participants were asked whether they had employed various self-help behaviours over the past month, using a single ‘check all that apply’ item (see online Supplementary Appendix Table E6 for a full list). These items were derived from a list of recommended strategies for self-managing mental health during COVID-19 from an Australian mental health support organisation (BeyondBlue, 2020).

#### Predictors

The primary predictor was time. For PHQ-9 and GAD-7 scores, there were two time points: August 2019−March 2020 (via the Wave 10 survey), and May−June 2020 (via the COVID-19 survey). Only Wave 10 responses up to 14 March 2020 (prior to the establishment of the National Cabinet on 15 March, and the commencement of COVID-19 restrictions in the following week), were included in the analysis. For help-seeking, there were two time points: February and May–June 2020 (both assessed via the COVID-19 survey).

A secondary predictor was gender (male/female). We controlled for various covariates measured in August 2019–March 2020: age, student status, employment, living status, area-level socioeconomic status, having older siblings, peer substance use and peer disapproval of substance use. The broader APSALS study focuses on adolescent alcohol use, therefore covariates were selected for their links with adolescent drinking and mental health (see online Supplementary Appendix C for further detail); we continued to include them given the strong links between alcohol use and mental health and potential shared covariate factors (e.g. Newton-Howes & Boden, 2016; Teesson *et al*., 2010).

### Statistical analysis

Characteristics of participants who completed the COVID-19 survey were compared to the broader APSALS cohort at Wave 10 (including those not invited to the survey) using *t* tests and χ^2^ tests. Participants' COVID-19 experiences were reported descriptively.

For Aim 1, we conducted analyses of change in PHQ-9 and GAD-7 scores pre- and during restrictions. We used mixed effects models to control for repeated observations for each participant, with linear models for raw scores (with results reported as a change in the mean), and logistic models for meeting the screening cut-off (with results reported as adjusted odds ratios). As a sensitivity analysis, we examined change in levels of severity using mixed effects ordinal logistic regression. Perceived change in mental health was studied descriptively.

For analysis of help-seeking (Aim 2), we also used mixed effects logistic regression, with two time-points: pre-restrictions (February 2020), and during restrictions (May–June 2020). We also report descriptive statistics for engagement in self-help behaviours.

For Aim 3, we reconducted all models with a gender-by-time interaction effect. As only two participants identified as a gender other than male or female, we excluded them from analysis. Analyses were conducted in Stata 14.1 (StataCorp, 2015) and R 3.6.3 (R Core Team, 2020). These analyses were not pre-registered and should be considered exploratory.

#### Missing data

To reduce the chance of bias due to missing data, we used multiple imputation, implemented using the R package ‘mice’ (van Buuren & Groothuis-Oudshoorn, 2011). Further detail is included in online Supplementary Appendix D.

## Results

### Sample characteristics

The COVID-19 subsample (*n* = 443) was generally similar to the broader APSALS cohort when assessed on Wave 10 characteristics but had a larger proportion of women (60% *v.* 51%; online Supplementary Table E1). Participants were aged on average 22.0 years (s.d. = 0.7) at the time of the COVID-19 survey. Two thirds (66.1%) reported currently living with their family, 21.5% with a partner, and 19.4% with housemates, and one in six (17%) reported a change in their living circumstances since restrictions (online Supplementary Table E2). The majority were studying full-time (February: 63.8%; May–June: 55.4%), and/or employed (February: 68.6%; May–June: 52.3%), with 8.8% reporting reduced pay and 25.6% reporting reduced hours of work since COVID-19 restrictions. Participants who completed the COVID-19 survey largely resided in Tasmania (37.5%), Western Australia (26.9%) and New South Wales (25.7%), reflecting the initial cohort recruitment jurisdictions (online Supplementary Table E3).

On completing the COVID-19 survey, 6.8% of respondents had been tested for COVID-19; one reported diagnosis. Three-quarters (75.1%) reported voluntary home isolation (i.e. staying home except for essential reasons such as work), and 9.2% reported having been in quarantine. Most (83.1%) reported being not at all or slightly worried about contracting COVID-19 (online Supplementary Table E4).

### Aim 1: Changes in mental health since COVID-19 restrictions, including perceived and prospective changes

#### Depression symptoms (PHQ-9)

There was a significant increase in mean scores for depression from before (*M* = 6.0, s.d. = 5.9) to during the pandemic (*M* = 7.2, s.d. = 6.2), after adjusting for covariates (coef 1.29; 95% CI 0.72–1.86; Table 1). Mean scores were above the ‘mild’ cut-off score at both time points (Table 2). Sensitivity analysis of severity categories was consistent with the primary analysis (online Supplementary Table E5).

**Table 1.** Predictors of depression and GAD score and of meeting cut-off scores for likely presence of depressive disorder and anxiety disorder during COVID-19 restrictions

		PHQ-9 Depression Coef (95% CI)	GAD-7 Anxiety Coef (95% CI)	Likely presence of depressive disorder (PHQ-9 > = 10) OR (95% CI)	Likely presence of anxiety disorder (GAD-7 > = 10) OR (95% CI)
Time	Aug 2019–Mar 2020	REF	REF	REF	REF
	May–June 2020	1.29 (0.72–1.86)	0.78 (0.26–1.31)	2.56 (1.54–4.28)	1.25 (0.78–2.01)
Gender	Male	REF	REF	REF	REF
	Female	2.96 (1.90–4.01)	2.85 (1.94–3.76)	7.23 (3.21–16.28)	5.77 (2.80–11.91)
Age		0.05 (−0.88 to 0.98)	0.02 (−0.79 to 0.83)	1.06 (0.58–1.93)	0.94 (0.54–1.61)
Student	No	REF	REF	REF	REF
	Yes	−0.52 (−1.63 to 0.59)	−0.08 (−1.05 to 0.89)	0.90 (0.44–1.81)	0.89 (0.47–1.69)
Employed	No	REF	REF	REF	REF
	Yes	−0.76 (−1.88 to 0.37)	−0.12 (−1.11 to 0.87)	0.66 (0.32–1.35)	0.86 (0.45–1.66)
Live alone	No	REF	REF	REF	REF
	Yes	−0.96 (−3.67 to 1.75)	−0.13 (−2.48 to 2.23)	0.31 (0.04–2.16)	0.84 (0.17–4.09)
SEIFA	Low	REF	REF	REF	REF
	Medium	−0.73 (−2.51 to 1.05)	−1.41 (−2.96 to 0.14)	0.73 (0.25–2.20)	0.59 (0.23–1.53)
	High	−1.29 (−2.88 to 0.30)	−1.85 (−3.24 to −0.46)	0.65 (0.24–1.73)	0.40 (0.17–0.95)
Older siblings	No	REF	REF	REF	REF
	Yes	0.32 (−0.70 to 1.34)	0.15 (−0.74 to 1.04)	1.31 (0.69–2.49)	1.38 (0.77–2.47)
Mean peer substance use (s.d.)		0.00 (−0.16 to 0.15)	0.00 (−0.14 to 0.14)	0.99 (0.90–1.10)	0.98 (0.90–1.08)
Mean peer disapproval of substance use (s.d.)		−0.28 (−0.65 to 0.09)	−0.26 (−0.58 to 0. 06)	0.85 (0.67–1.08)	0.82 (0.66–1.02)

*Note*. The primary exposure of interest was time. All covariates were measured August 2019–March 2020 (see online Supplementary Appendix C); coefficients and odds ratios are derived from multivariable mixed effects models. *SEIFA, Socio-Economic Indexes for Areas.

**Table 2.** Depression and GAD before and during the COVID-19 restrictions

		Mean (s.d.)/%	
		Aug 2019–Mar 2020	May–June 2020
Depression (PHQ-9)	Score	6.0 (5.9)	7.2 (6.2)
	Likely disorder	19.0%	28.5%
	None	49.4%	42.9%
	Minimal	31.6%	28.6%
	Mild	9.2%	14.5%
	Moderately severe	5.5%	8.4%
	Severe	4.3%	5.6%
Generalised Anxiety (GAD-7)	Score	5.0 (5.2)	5.7 (5.7)
	Likely disorder	17.9%	21.2%
	None	56.4%	51.7%
	Mild	25.7%	27.1%
	Moderate	11.5%	12.7%
	Severe	6.5%	8.5%

Table 2 shows that 19% of the sample reported symptoms at or above the clinical cut-off (i.e. indicative of further investigation for presence of depression) before the pandemic; this increased to 28.5% in May–June 2020. The odds of likely depressive disorder were significantly higher during the pandemic (2.56; 95% CI 1.54–4.28) compared to prior (Table 1).

#### Generalised Anxiety Disorder symptoms (GAD-7)

There was a significant increase in mean GAD scores from before (*M* = 5.0, s.d. = 5.2) to during the pandemic (*M* = 5.7, s.d. = 5.7), after controlling for covariates (coef 0.78; 95% CI 0.26–1.31; Table 1). Mean scores were above the cut-off for ‘mild’ generalised anxiety at both time points (see Table 2). Sensitivity analysis of severity categories was consistent with the primary analysis (online Supplementary Table E4).

Table 1 shows that there was no significant increase in the odds of meeting the clinical cut-off score for likely GAD from before to during the pandemic (17.9% *v.* 21.2%; OR 1.25; 95% CI 0.78–2.01).

#### Self-rated mental health

Just under half (44.9%) of the participants rated their mental health as poor or fair at May–June 2020, with the most common ratings being fair (31.6%) or good (29.8%; see online Supplementary Table E4). Half (49.3%) of the respondents reported their mental health was worse in May–June than it had been in February 2020, but a small proportion (11.6%) reported their mental health had improved since February (online Supplementary Table E4).

### Aim 2: Changes in help-seeking and self-help behaviours since COVID-19 restrictions

#### Changes in help-seeking

We did not find significant change in reported mental health help-seeking from health professionals between February 2020 (20.3%) and May–June 2020 (16.4%; OR: 0.50, 95% CI 0.19–1.32; Table 3).

**Table 3.** Predictors of change in seeking help for mental health during COVID-19 restrictions

		Sought help for mental health OR (95% CI)
Time	Aug 2019–Mar 2020	REF
	May–June 2020	0.50 (0.19–1.32)
Gender	Male	REF
	Female	2.35 (0.74–7.47)
Age		1.03 (0.37–2.82)
Student	No	REF
	Yes	1.13 (0.35–3.66)
Employed	No	REF
	Yes	1.19 (0.35–4.06)
Live alone	No	REF
	Yes	1.47 (0.09–23.14)
SEIFA	Low	REF
	Medium	0.56 (0.09–3.31)
	High	0.35 (0.07–1.74)
Older siblings	No	REF
	Yes	1.43 (0.49–4.15)
Mean peer substance use (s.d.)		0.91 (0.77–1.07)
Mean peer disapproval of substance use (s.d.)		0.67 (0.44–1.04)

*Note*. The primary exposure of interest was time. All covariates were measured August 2019–March 2020 (see online Supplementary Appendix C); coefficients and odds ratios are derived from multivariable mixed effects models.

#### Rates of self-help behaviours

Nearly all (99%) respondents reported they had performed at least one of the self-help behaviours (online Supplementary Table E6), the most common being staying connected with friends, family and colleagues via email, social media or phone (82.6%), getting regular exercise (69.2%), and reminding oneself this period of restrictions is temporary (66.9%).

### Aim 3: Changes in mental health and help-seeking since COVID-19 restrictions by gender

#### Prospective change in mental health

**Fig. 1.**
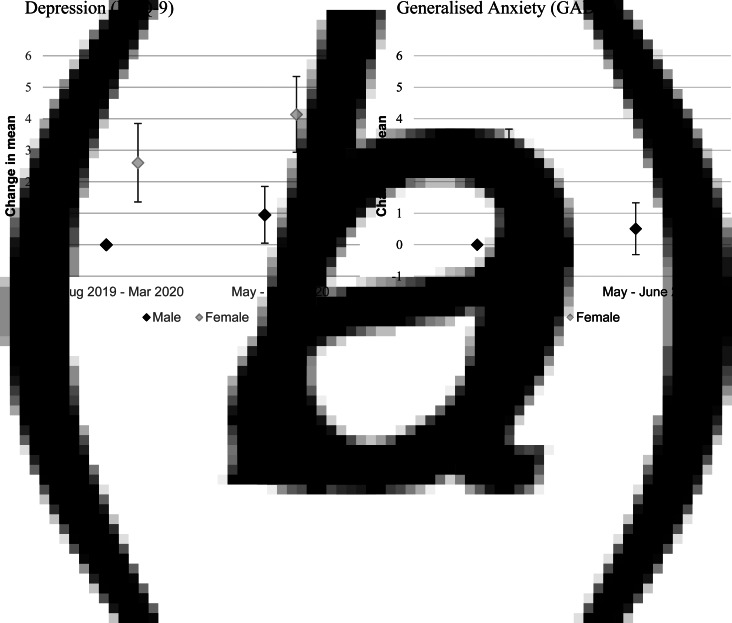
Change in depression and GAD score during COVID-19 restrictions compared to APSALS Wave 10 – by gender. *Note*: models are adjusted for covariates: age, gender, student status, employment status, whether participant lived alone, SEIFA, older siblings, peer substance use and peer disapproval of substance use. Full results are included in online Supplementary Table E6. PHQ-9 scores ranged from 0 to 27; GAD-7 scores ranged from 0 to 21.

Women reported higher levels of depression and generalised anxiety symptoms than men before the pandemic and since it commenced (Fig. 1). Both had increases in depression, but not GAD scores over this period (Fig. 1, online Supplementary Table E7). Sensitivity analyses for depression and generalised anxiety severity were similar, except that men did not show higher risk of increased depression severity over time (online Supplementary Fig. E1, Table E8). While women showed greater odds than men of meeting the cut-off score for depression (PHQ-9) and GAD (GAD-7) in both time periods, neither gender showed a significant increase in the odds of meeting the cut-off score for depression or GAD during the pandemic (online Supplementary Fig. E2, Table E9).

#### Changes in help-seeking

**Fig. 2.**
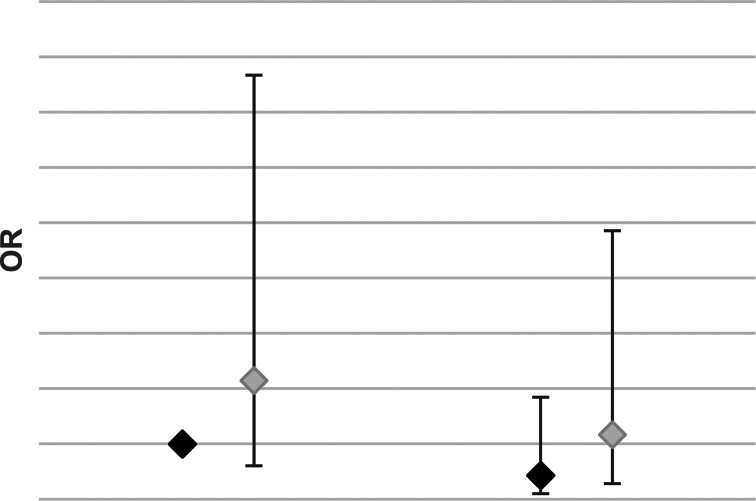
Change in seeking help for mental health – by gender. *Note*: model is adjusted for covariates. Full results are included in online Supplementary Table E9.

There were no differences by gender in reported changes to help-seeking from before to during the pandemic (Fig. 2 and online Supplementary Table E10).

## Discussion

We prospectively examined the impact of the COVID-19 pandemic and its associated restrictions on the mental health of young adults, showing that depression and generalised anxiety increased during restrictions, and that half the sample rated their mental health as having worsened. Increases in depression were accompanied by higher odds of meeting the clinical cut-off score for likely depressive disorder, with a third of the sample reporting symptoms at or above the cut-off score in May–June 2020. We did not observe changes in levels of mental health help-seeking from health professionals, although self-help behaviours during restrictions were commonly reported. Finally, we did not find that men and women differed in the extent to which their depression and generalised anxiety worsened during the pandemic. Together, these findings indicate change in mental health among young people during the COVID-19 pandemic, particularly an increase in depression symptoms, in the absence of increases in help-seeking.

The increase in depression and generalised anxiety scores in young people in this study align with evidence of the high prevalence of these symptoms in the general adult population (e.g. Newby, O'Moore, Tang, Christensen, & Faasse, 2020; Xiong *et al*. 2020), and internationally among young adults (e.g. Li, Cao, Leung, & Mak, 2020; Liu *et al*. 2020), since the pandemic. Participants' retrospective self-ratings of their mental health appeared to align with this prospective change, with half of participants reporting their mental health had worsened since February. Together, these findings reinforce that young Australians have experienced adverse mental health impacts in the early months of pandemic, which may translate to increased mental health burden. This may apply particularly to depression rather than GAD symptoms, as while symptoms increased for both disorders, there was only a higher likelihood of meeting the clinical threshold for depression.

It should be noted that levels of anxiety and depression symptomatology in this sample, while higher than pre-pandemic levels, appear lower than those observed among young adults during the pandemic in some studies in Western countries with higher rates of COVID-19 cases and deaths such as the US (e.g. Liu et al., 2020). This may suggest a unique impact in the Australian context commensurate with the severity of the COVID-19 crisis in this location. Further monitoring is warranted to determine whether symptomatology changes as the pandemic unfolds, particularly as restrictions on social distancing lift but support initiatives implemented for the pandemic (e.g. governmental financial support) cease.

However, it is important to acknowledge the diversity in responses: two-in-five participants reported no change and one-in-ten reported an improvement in their mental health since COVID-19 restrictions. These results may indicate higher resiliency for some individuals, which is further supported by high engagement in self-help behaviours among the samples. Although we were not able to examine their prospective relationship with mental health symptomatology in this study, some similar self-help strategies have been associated with increased resiliency in young adults during COVID-19 in one study internationally (Shanahan et al., 2020). Indeed, the self-help behaviours with the highest endorsement in this sample, seeking social support (82.9%) and getting regular exercise (69.2%) have both been associated with lower incidence of depression and anxiety symptoms during COVID-19 in other young adult samples (Liu et al., 2020; Zhang, Zhang, Ma, & Di, 2020). Further, emerging research has demonstrated incidences of post-traumatic growth attributed to the pandemic, in which some individuals display improvements in psychological functioning after facing adversity (Chi et al., 2020; Tomaszek & Muchacka-Cymerman, 2020). While we were not able to make causal inferences as to factors explaining changes in depression and anxiety symptomatology in this study, further research examining protective and risk factors for coping in young people will help inform intervention targets as the pandemic unfolds. For example, early research suggests that distress tolerance (Liu et al., 2020) and social support (Lee et al., 2020; Shanahan et al., 2020) are key intervention targets in this age group during the pandemic. Alternatively, results may suggest it is too early in the pandemic to fully observe changes in participants' mental health; thus, it is important to monitor changes in symptomatology longer term.

Despite increases in generalised anxiety and depression, there was no increased uptake of mental health help-seeking from health professionals observed. These findings align with research conducted before the pandemic showing low rates of mental health help-seeking among young people compared with other age-groups (Gulliver et al., 2010; Wilson, 2010), as well as one recent study showing continued low rates of psychological help-seeking in Chinese college students during the pandemic (Liang et al., 2020). Indeed, there is evidence that, in this population, higher psychological distress is associated with stronger intentions to *not* seek help from others (Wilson, 2010). The preference for self-reliance to solve mental health problems has been shown to be a key barrier to young adults seeking help (Gulliver et al., 2010), and may relate to fear of stigma which is another key barrier to help-seeking in this age group (Eisenberg, Downs, Golberstein, & Zivin, 2009; Gulliver et al., 2010). The high rates of self-help behaviours in the current sample, whilst encouraging, may also reflect a pattern of help-avoidance and self-reliance.

Alternatively, it is possible that participants in this sample who experienced increased symptoms of depression and anxiety found self-help strategies sufficient in managing their mental health, negating the need for external professional support. Indeed, despite significant increases from pre-pandemic levels, the majority of participants reported GAD and depression symptoms within the lower (Mild) ranges during the pandemic, which may not have reached sufficient severity to prompt external help-seeking. This may apply particularly for GAD symptoms where odds of meeting the clinical threshold did not increase. This may account for lack of change in external help-seeking rates but high rates of engagement in self-help behaviours in this sample. Indeed the willingness to use self-help strategies, as well as external help-seeking, are both key components of mental health literacy in young people (Riebschleger, Costello, Cavanaugh, & Grové, 2019). However, without longitudinal data it is difficult to draw conclusions regarding the impact of these self-help strategies on anxiety and depression symptomatology.

Conversely, the finding that help-seeking did not increase during the pandemic despite arguably increased need for support may suggest that some restrictions, such as social distancing, have formed new barriers to treatment access (Power et al., 2020). Although the Australian Government introduced initiatives to increase access to mental health support during the pandemic, including increased telehealth and online support (Department of Health, 2020), there may be a lag in young Australians accessing this support. Cost is a key barrier to treatment access in young people (Gulliver et al., 2010). Reduction in income during the pandemic may be a factor in continued low rates of help-seeking: while government rebates are available, for example, these do not cover the entire cost of psychological treatment. There have also been longer than usual wait-times for psychological treatment (Academic Unit of General Practice, 2020). As the pandemic unfolds and demands on services increase, it is critical to increase accessibility to psychological treatment for young people. This may include further government subsidisation of psychological treatment and increasing the mental health professional workforce capacity; it is also important to promote awareness of current rebates as well as the numerous evidence-based free or low-cost online treatment clinics available in Australia. Continuing efforts by government agencies and health providers to provide accessible psychoeducation about mental health and self-help strategies will also be important, particularly in reducing stigma (Riebschleger et al., 2019).

Efforts to target young people for mental health support may need to consider gender differences. Women displayed higher depression and generalised anxiety scores across both time points, although there was no evidence of a differential change in these outcomes by gender pre- *v.* during COVID-19 restrictions. Further, despite a higher burden of anxiety and depression symptoms in females in this cohort, there were no gender differences in help-seeking rates. These findings align with literature showing that females are particularly vulnerable to these mental health problems (Leach et al., 2008; Piccinelli & Wilkinson, 2000), including during the pandemic (Torales et al., 2020; Xiong et al., 2020). They contrast however with research showing females have higher rates of help-seeking for psychological problems than men (e.g. Galdas, Cheater, & Marshall, 2005; Liddon, Kingerlee, & Barry, 2018). The unique nature of the COVID-19 pandemic and associated restrictions may in part explain this discrepancy relative to research conducted prior to the pandemic, and requires exploring in future research. Whilst we did not see differential mental health impacts of COVID-19 between males and females in this study, it is important to monitor these outcomes longer-term.

### Strengths and limitations

This study is the first to our knowledge to concentrate on quantifying the impact of the COVID-19 pandemic and associated restrictions on the mental health of young Australian adults. Key strengths were the use of longitudinal data and validated scales for primary outcomes. Participants were asked to recall help-seeking behaviours during February and thus, recall bias should be acknowledged as a potential limitation. The self-help measure used check-all response options, which can elicit less deep thinking than forced-choice questions for each behaviour (Smyth, Dillman, Christian, & Stern, 2006). The addition of clinician-rated measures of mental health would have strengthened measurement but were not feasible given the nature of survey administration. A further limitation is the opt-in nature of recruitment for the original cohort and the COVID survey, although the sample for the latter was broadly similar to the wider cohort. The APSALS cohort is generally representative of the wider Australian population of the same age group but may overrepresent young adults of higher socioeconomic status (Aiken et al., 2017). It is worth noting that ethnicity was not measured however participating parent country of birth (73.8% Australia) was similar to the overall Australian population at time of recruitment (Aiken et al., 2017). Finally, while we have adjusted for a range of covariates, the APSALS study was designed with a focus on alcohol, so it is possible that changes in mental health were related to other factors not measured in the study.

## Conclusions

This study provides prospective evidence of increased depression and generalised anxiety symptoms, with no corresponding increase in treatment-seeking, in a sample of young adults since the COVID-19 pandemic in Australia. These results support assertions that the pandemic and associated restrictions have had an adverse impact on the mental health of young adults. It is important to reduce barriers to support including increasing the mental health workforce capacity, ongoing subsidisation of psychological services, and promotion of existing rebates and free online treatment and psychoeducation resources available. Ongoing monitoring of mental health outcomes and treatment-seeking behaviour is warranted given that the full impact of the pandemic may not yet be realised. This includes identifying modifiable risk and protective factors for mental health and treatment-seeking outcomes among young people, to inform psychological interventions targeted to this age group, and address the potentially widening gap between those needing and accessing treatment.

## Footnotes

† The notes appear after the main text.

1 In addition to federal restrictions, additional state government-imposed restrictions varied across Australia according to changing case numbers and locations. See online Supplementary Appendix A (Fig. A2) for an illustration of the stringency of Australia's response to COVID-19 at the time of survey completion.

## Supplementary material

For supplementary material accompanying this paper visit http://dx.doi.org/10.1017/S0033291721001963. click here to view supplementary material

## Clinical Trial Registration

ClinicalTrials.gov (NCT02280551)
