# Supplementary material for: Changes in mental health and help-seeking among young Australian adults during the COVID-19 pandemic: a prospective cohort study
Source: Psychol Med. 2021 May 10:1–9. doi: 10.1017/S0033291721001963 (PMC8144825; doi:10.1017/S0033291721001963)
Supplement: Supplementary file 1 [file S0033291721001963sup001.docx]

# Mental health and help-seeking among young Australian adults during the COVID-19 pandemic: a prospective cohort study – Appendices

[Appendix A – Timeline of survey completion 2](#_Toc50374334)

[Appendix B – STROBE 4](#_Toc50374335)

[Appendix C – Measures 7](#_Toc50374336)

[Appendix D – Missing data 11](#_Toc50374337)

[Appendix E – Additional Results 13](#_Toc50374338)

## Appendix A – Timeline of survey completion

**Figure A1** Dates of survey completion shown against COVID-19 cases in Australia

Note: Dates of COVID-19 survey completion shown as blue line. Number of daily cases and cumulative cases from covid19data.com.au (Ambrogetti, Gilbert, & Janif, 2020).

**Figure A2** Dates of survey completion shown against stringency of Australian response to COVID-19

Note: Dates of survey completion shown as blue line. Stringency from Coronavirus Government Response Tracker (Hale et al., 2020). Cumulative cases from covid19data.com.au (Ambrogetti et al., 2020).

## Appendix B – STROBE

**Table B1** STROBE checklist

|  | Item No. | Recommendation |  |
| --- | --- | --- | --- |
| **Title and abstract** | 1 | (*a*) Indicate the study’s design with a commonly used term in the title or the abstract | ✓ |
|  |  | (*b*) Provide in the abstract an informative and balanced summary of what was done and what was found | ✓ |
| Introduction | | | |
| Background/rationale | 2 | Explain the scientific background and rationale for the investigation being reported | ✓ |
| Objectives | 3 | State specific objectives, including any prespecified hypotheses | ✓ |
| Methods | | | |
| Study design | 4 | Present key elements of study design early in the paper | ✓ |
| Setting | 5 | Describe the setting, locations, and relevant dates, including periods of recruitment, exposure, follow-up, and data collection | ✓ |
| Participants | 6 | (*a*) Give the eligibility criteria, and the sources and methods of selection of participants. Describe methods of follow-up | ✓ published elsewhere |
|  |  | (*b*) For matched studies, give matching criteria and number of exposed and unexposed | N/A |
| Variables | 7 | Clearly define all outcomes, exposures, predictors, potential confounders, and effect modifiers. Give diagnostic criteria, if applicable | ✓ |
| Data sources/ measurement | 8* | For each variable of interest, give sources of data and details of methods of assessment (measurement). Describe comparability of assessment methods if there is more than one group | ✓ |
| Bias | 9 | Describe any efforts to address potential sources of bias | ✓ |
| Study size | 10 | Explain how the study size was arrived at | N/A |
| Quantitative variables | 11 | Explain how quantitative variables were handled in the analyses. If applicable, describe which groupings were chosen and why | ✓ |
| Statistical methods | 12 | (*a*) Describe all statistical methods, including those used to control for confounding | ✓ |
|  |  | (*b*) Describe any methods used to examine subgroups and interactions | ✓ |
|  |  | (*c*) Explain how missing data were addressed | ✓ |
|  |  | (*d*) If applicable, explain how loss to follow-up was addressed | ✓ |
|  |  | (*e*) Describe any sensitivity analyses | ✓ |
| Results | | | |
| Participants | 13* | (a) Report numbers of individuals at each stage of study—eg numbers potentially eligible, examined for eligibility, confirmed eligible, included in the study, completing follow-up, and analysed | ✓ |
|  |  | (b) Give reasons for non-participation at each stage | ✓ |
|  |  | (c) Consider use of a flow diagram | ✓ |
| Descriptive data | 14* | (a) Give characteristics of study participants (eg demographic, clinical, social) and information on exposures and potential confounders | ✓ |
|  |  | (b) Indicate number of participants with missing data for each variable of interest | ✓ |
|  |  | (c) Summarise follow-up time (eg, average and total amount) | ✓ |
| Outcome data | 15* | Report numbers of outcome events or summary measures over time | ✓ |
| Main results | 16 | (*a*) Give unadjusted estimates and, if applicable, confounder-adjusted estimates and their precision (eg, 95% confidence interval). Make clear which confounders were adjusted for and why they were included | ✓ |
|  |  | (*b*) Report category boundaries when continuous variables were categorized | N/A |
|  |  | (*c*) If relevant, consider translating estimates of relative risk into absolute risk for a meaningful time period | N/A |
| Other analyses | 17 | Report other analyses done—eg analyses of subgroups and interactions, and sensitivity analyses | ✓ |
| Discussion | | | |
| Key results | 18 | Summarise key results with reference to study objectives | ✓ |
| Limitations | 19 | Discuss limitations of the study, taking into account sources of potential bias or imprecision. Discuss both direction and magnitude of any potential bias | ✓ |
| Interpretation | 20 | Give a cautious overall interpretation of results considering objectives, limitations, multiplicity of analyses, results from similar studies, and other relevant evidence | ✓ |
| Generalisability | 21 | Discuss the generalisability (external validity) of the study results | ✓ |
| Other information |  | | |
| Funding | 22 | Give the source of funding and the role of the funders for the present study and, if applicable, for the original study on which the present article is based | ✓ |

**Figure B1** Study flowchart of recruitment and assessment of APSALS cohort

| **2010-2011** |  | **Recruitment**  49 Grade 7 year groups in Sydney, Hobart, Perth  5,759 families approached  2,017 families agreed to opt-in  1,927 adolescent-parent dyads eligible | \|  \| \| --- \| | 90 ineligible:  16 adolescents were not in year Grade 7  74 parents did not send signed consent |
| --- | --- | --- | --- | --- | --- |
|  | | | | |
| **2010-2011**  M=12.9 years |  | **Wave 1 (Grade 7) respondents**  1,906 adolescent-parent dyad responses **98.9%** | \|  \| \| --- \| | 9 revoked consent  12 lost to follow-up |
|  | | | | |
| **2011-2012**  M=13.9 years |  | **Wave 2 (Grade 8) respondents**  1,823 adolescent-parent dyad responses **94.6%** | \|  \| \| --- \| | 9 revoked consent  2 lost to follow-up |
|  | | | | |
| **2012-2013**  M=14.8 years |  | **Wave 3 (Grade 9) respondents**  1,755 adolescent-parent dyad responses **99.1%** | \|  \| \| --- \| | 24 revoked consent  9 lost to follow-up |
|  | | | | |
| **2013-2014**  M=15.8 years |  | **Wave 4 (Grade 10) respondents**  1,691 adolescent-parent dyad responses **87.8%** | \|  \| \| --- \| | 8 revoked consent  0 lost to follow-up |
|  | | | | |
| **2014-2015**  M=16.9 years |  | **Wave 5 (Grade 11) respondents**  1,657 adolescent-parent dyad responses **86.0%** | \|  \| \| --- \| | 2 revoked consent  9 lost to follow-up |
|  | | | | |
| **2015-2016**  M=17.8 years |  | **Wave 6 (Grade 12) respondents**  1,611 adolescent responses **83.6%** | \|  \| \| --- \| | 10 revoked consent  20 lost to follow-up |
|  | | | | |
| **2016-2017**  M=18.8 years |  | **Wave 7 respondents**  1,494 adolescent responses **77.5%** | \|  \| \| --- \| | 9 revoked consent  8 lost to follow-up |
|  | | | | |
| **2017-2018**  M=19.8 years |  | **Wave 8 respondents**  1,427 adolescent responses **74.1%** | \|  \| \| --- \| | 13 revoked consent  1 lost to follow-up |
|  | | | | |
| **2018-2019**  M=20.8 years |  | **Wave 9 respondents**  1,561 adolescent responses **81.0%** | \|  \| \| --- \| | 9 revoked consent  1 lost to follow-up |
|  | | | | |
| **2019-2020**  M=21.7 years |  | **Wave 10 respondents***  1,180 adolescent responses **61.2%** | \|  \| \| --- \| | 2 revoked consent  0 lost to follow-up |
|  | | | | |
|  |  | **COVID-19 Survey respondents**  443 adolescent responses **77.5% Cohort 54.5% of those invited completed survey** | \|  \| \| --- \| |  |

Note: Wave 10 had not yet been completed at the time of the COVID-19 survey.

## Appendix C – Measures

A summary of the outcome measures, including the waves for which each was asked and included, can be seen in Table C1.

Descriptive demographics measures, including experiences with COVID-19

*Demographics questions.* Participants were asked a range of demographics questions in the APSALS survey (age, gender; see covariates section below) and in the COVID-19 survey (employment status and living situation, and whether these had changed since COVID-19 restrictions).

*Experiences with COVID-19.* Participants were asked questions related to their experience of COVID-19 including whether they had been diagnosed with the virus or completed voluntary home isolation or quarantine.

*Worry about COVID-19.* Participants were asked to rate how worried they were about contracting COVID-19 on a five-point Likert scale from *Not at all* to *Extremely*.

### Covariate measures

The APSALS study is concentrated on adolescent alcohol use behaviours, specifically on how parental supply of alcohol influences different patterns of alcohol consumption and related harms. Covariates captured in the APSALS surveys were chosen based on the literature on adolescent drinking and adolescent mental health. We continued to include them in the current study given the strong links between alcohol use and mental health and potential shared covariate factors (e.g., Newton-Howes & Boden, 2016; Teesson et al., 2010). Variables included here were measured from August 2019-March 2020. The variables included were: child gender and age (Fisher, Miles, Austin, Camargo, & Colditz Jr, 2007; Swendsen et al., 2012), socio-economic status of area of residence (Pink, 2008), having older siblings (Fisher et al., 2007), and their peer’s substance use and disapproval of alcohol and tobacco use (Fisher et al., 2007; Kuperman et al., 2013; Swendsen et al., 2012).

*Child gender.* Participants were given the opportunity to identify as male, female, or another gender. Because only n=2 participants identified as other than male or female, they were excluded from the analysis.

*Child age.* Age was included as a continuous variable, based on the child’s age in years (with months and days decimalised) at the time of survey completion.

*Socioeconomic status (SES).* SES was measured using Socio-Economic Indexes for Areas (SEIFA) Index of Relative Socio-Economic Advantage/Disadvantage (IRSAD), a population measure standardized to a mean of 1,000 (Pink, 2008); the mean score in the present sample was 1,025·1 (SD = 79·7) (Statistics, 2006). This was calculated based on the postcode of the area of residence, provided at wave 1. The IRSAD score is calculated using Australian national census data and a wide range of socioeconomic variables are used in the construction of the index including household income, educational levels, occupational levels, rent and mortgage repayments, dwelling size and number of occupants and disability rates within the area (Pink, 2008).

*Older siblings.* Parents were asked if there were any older siblings in the family, other than the child participating in the study.

*Peer use of alcohol and/or tobacco.* This was adapted from the 2011 Monitoring the Future (MTF) survey (Johnston, 1980). Six items asked adolescents about their peers’ quantity and frequency of alcohol consumption, and smoking tobacco. Each item had five response options, ranging from none to all (of their peers). Items were summed (score range: 6-30), and higher scores indicated higher levels of peer alcohol and/or tobacco use.

*Peer disapproval of alcohol and/or tobacco use.* Another four items from the 2011 MTF survey were also used (Johnston, 1980). Adolescents were asked how they thought their close friends would feel about them: smoking cigarettes, consuming any alcohol, consuming 1-2 drinks daily, and binge drinking over weekends. Each item had three response options: not disapprove, disapprove, and strongly disapprove. These items were summed (score range: 4-12), with a higher score indicating more peer disapproval of substance use.

**Table C1** Summary of times at which each outcome measure was assessed

|  | APSALS Main Survey | | COVID-19 Survey | |
| --- | --- | --- | --- | --- |
|  | Wave 10 (Aug 2019 - March 2020) | | Feb 2020 (retrospective) | May - June 2020 |
| PHQ-9 (Depression) | ✓ | |  | ✓ |
| GAD-7 (Generalised Anxiety) | ✓ | |  | ✓ |
| Self-rated mental health and perceived change since February |  |  |  | ✓ |
| Help-seeking behaviours (from health professionals) |  | | ✓ | ✓ |
| Self-help behaviours relevant to COVID-19 |  |  |  | ✓ |

### References

Ambrogetti, T., Gilbert, R., & Janif, A. (2020). Coronavirus (COVID-19) in Australia. Retrieved from <https://www.covid19data.com.au/>

Fisher, L. B., Miles, I., Austin, S., Camargo, C. A., & Colditz Jr, G. A. (2007). Predictors of initiation of alcohol use among US adolescents: findings from a prospective cohort study. *Archives of Pediatrics and Adolescent Medicine, 161*(10), 959-966. doi:10.1001/archpedi.161.10.959

Graham, J. W., Olchowski, A. E., & Gilreath, T. D. (2007). How many imputations are really needed? Some practical clarifications of multiple imputation theory. *Prevention Science, 8*(3), 206-213. doi:10.1007/s11121-007-0070-9

Hale, T., Angrist, N., Cameron-Blake, E., Hallas, L., Kira, B., Majumdar, S., . . . Webster, S. (2020). *Oxford COVID-19 Government Response Tracker*. Retrieved from

Hughes, R. A., Heron, J., Sterne, J. A. C., & Tilling, K. (2019). Accounting for missing data in statistical analyses: multiple imputation is not always the answer. *International Journal of Epidemiology, 48*(4), 1294-1304. doi:10.1093/ije/dyz032

Huque, M. H., Carlin, J. B., Simpson, J. A., & Lee, K. J. (2018). A comparison of multiple imputation methods for missing data in longitudinal studies. *BMC medical research methodology, 18*(1), 168. doi:10.1186/s12874-018-0615-6

Johnston, L. D. (1980). *Monitoring the Future: Questionnaire Responses from the Nation's High School Seniors, 1979*: ERIC.

Kuperman, S., Chan, G., Kramer, J. R., Wetherill, L., Bucholz, K. K., Dick, D., . . . Schuckit, M. (2013). A model to determine the likely age of an adolescent’s first drink of alcohol. *Pediatrics, 131*(2), 242-248. doi:10.1542/peds.2012-0880

Newton-Howes, G., & Boden, J. M. (2016). Relation between age of first drinking and mental health and alcohol and drug disorders in adulthood: evidence from a 35-year cohort study. *Addiction, 111*(4), 637-644. doi:<https://doi.org/10.1111/add.13230>

Pink, B. (2008). Information paper: an introduction to socio-economic indexes for areas (SEIFA), 2006. *Canberra: Australian Bureau of Statistics (ABS)*.

Statistics, A. B. o. (2006). Socio-economic indexes for areas (SEIFA), postal area (POA) Index of Relative Socio-Economic Advantage and Disadvantage, data cube; 2008. In: Author. Canberra,, Australia.

Swendsen, J., Burstein, M., Case, B., Conway, K. P., Dierker, L., He, J., & Merikangas, K. R. (2012). Use and abuse of alcohol and illicit drugs in US adolescents: results of the national comorbidity survey–adolescent supplement. *Archives of General Psychiatry, 69*(4), 390-398. doi:10.1001/archgenpsychiatry.2011.1503

Teesson, M., Hall, W., Slade, T., Mills, K., Grove, R., Mewton, L., . . . Haber, P. (2010). Prevalence and correlates of DSM-IV alcohol abuse and dependence in Australia: findings of the 2007 National Survey of Mental Health and Wellbeing. *Addiction 105*(2), 2085-2094.

van Buuren, S., & Groothuis-Oudshoorn, K. (2011). mice: multivariate imputation by chained equations in R. *Journal of Statistical Software, 45*(3). doi:10.18637/jss.v045.i03

## Appendix D – Missing data

The data contained a number of missing data points, both because not all participants completed all three surveys used in the analysis, and also because participants could refuse to answer individual questions in the surveys. Data was confirmed to be not missing completely at random via Little’s test. As such, we have assumed the data to be missing at random. While there was relatively little missing data, because missingness can introduce bias when there is missingness in both the outcome and exposure variables (Hughes, Heron, Sterne, & Tilling, 2019), the analyses were conducted using multiple imputation. Based on past research (Huque, Carlin, Simpson, & Lee, 2018), imputation conducted via the ‘just another variable’ approach, in which data is imputed in ‘wide’ form, with one record per individual, and each repeated measurement of the same variable imputed as a separate variable.

We conducted the imputation in the R package ‘mice’ (van Buuren & Groothuis-Oudshoorn, 2011). Just under a quarter of cases had at least some missing data (23%). Frequencies and the most common patterns of missing data are reported in Table D1. To be conservative, we used M=50 imputations (Graham, Olchowski, & Gilreath, 2007). Imputation models included all variables included in the analysis, as well as auxiliary variables related to mental health, use of services, and personal situation (e.g., employment status, living situation). Analyses were then conducted on each imputed dataset, and combined using Rubin’s rules.

**Table D1** Patterns of missing data

| **Wave** | **Variable** | **Most common patterns of missing data** | | | | | | | **Number missing in variable** |
| --- | --- | --- | --- | --- | --- | --- | --- | --- | --- |
|  |  | **(1)** | **(2)** | **(3)** | **(4)** | **(5)** | **(6)** | **(7)** |  |
| May-June 2020 (COVID-19 Survey) | PHQ Score |  |  |  |  | X | X |  | 30 |
|  | GAD Score |  |  |  |  | X | X |  | 27 |
|  | Self-rated mental health |  |  |  |  | X |  |  | 20 |
|  | Perceived change in mental health |  |  |  |  | X |  |  | 19 |
|  | Sought help for mental health |  |  |  |  | X | X |  | 25 |
|  | Reminded self restrictions were temporary |  |  |  |  | X |  |  | 24 |
|  | Tried to find reliable information |  |  |  |  | X |  | X | 31 |
|  | Stayed connected with friends etc |  |  |  |  | X |  | X | 31 |
|  | Engaged in healthy activities |  |  |  |  | X |  | X | 31 |
|  | Kept regular sleep routines |  |  |  |  | X |  | X | 31 |
|  | Tried to regularly get exercise |  |  |  |  | X |  | X | 31 |
|  | Avoided distressing news and social media |  |  |  |  | X |  | X | 31 |
|  | Sought support from friends and family |  |  |  |  | X |  | X | 31 |
|  | Live with family |  |  |  |  |  |  |  | 2 |
|  | Live with partner |  |  |  |  |  |  |  | 2 |
|  | Live with others (e.g., housemates) |  |  |  |  |  |  |  | 2 |
|  | Living situation the same as February 2020 |  |  |  |  |  |  |  | 2 |
|  | Student |  |  |  |  | X |  |  | 7 |
|  | Employed |  |  |  |  | X |  |  | 7 |
|  | Tested/diagnosed for COVID-19 |  |  |  |  | X |  |  | 21 |
|  | Worry about COVID-19 |  |  |  |  | X |  |  | 25 |
|  | Self-isolated |  |  |  |  | X |  |  | 27 |
|  | Quarantined |  |  |  |  | X |  |  | 27 |
| Feb 2020 (COVID-19 Survey) | Sought help for mental health |  |  |  |  | X | X |  | 25 |
|  | Student |  |  |  |  | X |  |  | 7 |
|  | Employed |  |  |  |  | X |  |  | 7 |
| Aug 2019-Mar 2020 (Wave 10) | PHQ Score |  |  |  | X |  |  |  | 8 |
|  | GAD Score |  |  |  | X |  |  |  | 8 |
| Sep 2018 - May 2019 (Wave 9) | Gender |  |  |  |  |  |  |  | 2 |
|  | Income | X |  |  |  |  |  |  | 24 |
|  | Age |  |  |  |  |  |  |  | 2 |
|  | Peer use of substances |  |  |  |  |  |  |  | 2 |
|  | Peer disapproval of substance use |  |  |  |  |  |  |  | 2 |
|  | SEIFA |  |  |  |  |  |  |  | 2 |
|  | Family history of alcohol problems |  | X |  |  |  |  |  | 20 |
|  | Have older siblings |  |  |  |  |  |  |  | 2 |
| Number missing in pattern | | 18 | 16 | 14 | 7 | 5 | 5 | 5 |  |

## Appendix E – Additional Results

**Table E1** Comparison of COVID-19 survey subsample to whole sample in wave 10

|  |  | **Full sample, Wave 10**  **(n=1180)** | **COVID Survey Subsample**  **(n=443)** | **Statistic; p-value** |
| --- | --- | --- | --- | --- |
| Mean age (SD) |  | 21.7 (0.5) | 21.7 (0.5) | t(1177)=1.78; p=0.075 |
| Gender | Male | 48.8% | 39.5% | χ2(1)=22.92; p<0.001 |
|  | Female | 51.2% | 60.5% |  |
| Income | $1-$12,999 | 22.2% | 25.4% | χ2(3)=7.52; p=0.057 |
|  | $13,000-$31,199 | 39.2% | 38.9% |  |
|  | $31,200-$67,599 | 33.1% | 29.1% |  |
|  | $67,600+ | 5.6% | 6.6% |  |
| Single Parent Household | No | 49.9% | 51.0% | χ2(1)=0.28; p=0.598 |
|  | Yes | 50.1% | 49.0% |  |
| Older siblings* | No | 48.1% | 48.5% | χ2(1)=0.02; p=0.888 |
|  | Yes | 51.9% | 51.5% |  |
| Parent born in Australia* | No | 26.5% | 27.9% | χ2(1)=0.50; p=0.481 |
|  | Yes | 73.5% | 72.1% |  |
| Parent education* | High school or less | 31.7% | 26.3% | χ2(2)=15.77; p<0.001 |
|  | Diploma, Trade, non-trade | 32.1% | 29.7% |  |
|  | University degree | 36.3% | 44.0% |  |
| Parent religiousity* | Not/a little | 70.5% | 65.8% | χ2(1)=6.04; p=0.014 |
|  | Pretty/very | 29.5% | 34.2% |  |
| Parent employment* | Employed (full-time/part-time) | 81.1% | 83.4% | χ2(2)=2.30; p=0.317 |
|  | Unemployed - in workforce | 12.5% | 11.3% |  |
|  | Unemployed - not in workforce | 6.4% | 5.2% |  |
| Mean parent demandingness* (SD) | | 23.7 (3.7) | 23.7 (3.5) | t(1898)=-0.01; p=0.993 |
| Mean parent responsiveness* (SD) | | 29.7 (4.3) | 29.8 (4.3) | t(1899)=-0.48; p=0.634 |
| SEIFA* | Low | 17.3% | 13.8% | χ2(2)=5.57; p=0.062 |
|  | Medium | 21.9% | 21.3% |  |
|  | High | 60.8% | 64.9% |  |
| Mean peer substance use (SD) | | 14.6 (4.0) | 14.2 (4.1) | t(1164)=2.21; p=0.027 |
| Mean peer disapproval of substance use (SD) | | 2.2 (1.7) | 2.5 (1.7) | t(1164)=-3.66; p<0.001 |

Note. * Baseline variable. All other variables were assessed in Wave 10.

**Table E2** Occupation and living situation before and during the COVID-19 restrictions

|  |  | **%** | |
| --- | --- | --- | --- |
|  |  | **Aug 2019 - Mar 2020** | **May - June 2020** |
| Occupation | Student | 63.8% | 55.4% |
|  | Employed | 68.6% | 52.3% |
| Living situation | Live alone | 3.4% | 3.4% |
|  | Live with family | 63.7% | 66.1% |
|  | Live with partner | 97.5% | 21.5% |
|  | Live with others (eg housemates) | 24.2% | 19.4% |

**Table E3** Location of participants who completed COVID-19 survey

| **State** | **n (%)** |
| --- | --- |
| Australian Capital Territory (ACT) | 6 (1.4%) |
| New South Wales (NSW) | 114 (25.7%) |
| Queensland (QLD) | 13 (2.9%) |
| South Australia (SA) | 1 (0.2%) |
| Tasmania (TAS) | 166 (37.5%) |
| Victoria (VIC) | 24 (5.4%) |
| Western Australia (WA) | 119 (26.9%) |

**Table E4** Self-reported mental health and other mental health variables in the sample during the COVID-19 restrictions

|  |  | **May - June 2020 mean (sd) / %** |
| --- | --- | --- |
| Self-rated mental health during COVID-19 restrictions | Excellent | 6.2% |
|  | Very good | 19.1% |
|  | Good | 29.8% |
|  | Fair | 31.6% |
|  | Poor | 13.3% |
| Perceived change in mental health due to COVID-19 restrictions | Worse | 49.3% |
|  | Same | 39.1% |
|  | Better | 11.6% |
| Worry about COVID | Not at all | 43.4% |
|  | Slightly | 39.7% |
|  | Moderately | 14.3% |
|  | Very | 2.2% |
|  | Extremely | 0.5% |

**Table E5** Predictors of depression and GAD severity during COVID-19 restrictions

|  |  | **PHQ-9 Severity OR (95% CI)** | **GAD-7 Severity OR (95% CI)** |
| --- | --- | --- | --- |
| Time | Aug 2019-Mar 2020 | REF | REF |
|  | May-June 2020 | 2.13 (1.50, 3.01) | 1.46 (1.03, 2.08) |
| Gender | Male | REF | REF |
|  | Female | 5.00 (2.70, 9.28) | 5.62 (3.09, 10.21) |
| Age | | 0.97 (0.58, 1.62) | 1.00 (0.62, 1.63) |
| Student | No | REF | REF |
|  | Yes | 1.00 (0.55, 1.84) | 0.87 (0.49, 1.53) |
| Employed | No | REF | REF |
|  | Yes | 0.70 (0.38, 1.29) | 0.98 (0.55, 1.76) |
| Live alone | No | REF | REF |
|  | Yes | 0.82 (0.19, 3.53) | 1.08 (0.28, 4.23) |
| SEIFA | Low | REF | REF |
|  | Medium | 0.53 (0.21, 1.37) | 0.44 (0.18, 1.08) |
|  | High | 0.43 (0.18, 1.00) | 0.37 (0.17, 0.82) |
| Older siblings | No | REF | REF |
|  | Yes | 1.18 (0.68, 2.05) | 1.00 (0.60, 1.69) |
| Mean peer substance use (SD) | | 0.96 (0.89, 1.05) | 0.99 (0.91, 1.07) |
| Mean peer disapproval of substance use (SD) | | 0.81 (0.66, 1.00) | 0.85 (0.70, 1.03) |

**Table E6** Self-help behaviours enacted during COVID-19 restrictions

|  | **%** |
| --- | --- |
| Reminded self this period of restrictions is temporary | 66.9% |
| Tried to find reliable sources of information about COVID-19 (e.g., government websites) | 60.7% |
| Stayed connected with friends, family and colleagues via email, social media or phone | 82.6% |
| Engaged in healthy activities that you enjoy and find relaxing | 66.2% |
| Kept regular sleep routines | 43.4% |
| Tried to regularly get exercise | 69.2% |
| Avoided news and social media you find distressing | 47.1% |
| Sought support from friends and family when needed | 47.3% |

**Table E7** Predictors of depression and GAD score during COVID-19 restrictions – time by gender interaction

|  |  | **PHQ-9 Depression Coef (95% CI)** | **GAD-7 Anxiety Coef (95% CI)** |
| --- | --- | --- | --- |
| Time x Gender | Male - Aug 2019-Mar 2020 | REF | REF |
|  | Male - May-June 2020 | 0.95 (0.05, 1.85) | 0.51 (-0.32, 1.33) |
|  | Female - Aug 2019-Mar 2020 | 2.60 (1.36, 3.85) | 2.57 (1.47, 3.67) |
|  | Female - May-June 2020 | 4.14 (2.94, 5.34) | 3.55 (2.48, 4.61) |
| Age | | 0.04 (-0.88, 0.97) | 0.02 (-0.79, 0.83) |
| Student | No | REF | REF |
|  | Yes | -0.52 (-1.63, 0.59) | -0.08 (-1.05, 0.89) |
| Employed | No | REF | REF |
|  | Yes | -0.76 (-1.89, 0.37) | -0.12 (-1.11, 0.87) |
| Live alone | No | REF | REF |
|  | Yes | -0.95 (-3.66, 1.76) | -0.12 (-2.48, 2.24) |
| SEIFA | Low | REF | REF |
|  | Medium | -0.72 (-2.50, 1.06) | -1.40 (-2.95, 0.15) |
|  | High | -1.27 (-2.86, 0.32) | -1.83 (-3.22, -0.44) |
| Older siblings | No | REF | REF |
|  | Yes | 0.32 (-0.70, 1.35) | 0.16 (-0.73, 1.04) |
| Mean peer substance use (SD) | | 0.00 (-0.16, 0.15) | 0.00 (-0.14, 0.14) |
| Mean peer disapproval of substance use (SD) | | -0.28 (-0.65, 0.09) | -0.26 (-0.58, 0.06) |

**Figure E1** Change in severity of depression and GAD during COVID-19 restrictions compared to APSALS Wave 10 – time by gender interaction

| (a) Depression (PHQ-9) | (b) Generalised Anxiety (GAD-7) |
| --- | --- |
|  |  |

Note: ORs shown on a log scale. Models are adjusted for covariates. Full results are included in Supplementary Table E7.

**Table E8** Predictors of depression and GAD severity during COVID-19 restrictions – time by gender interaction

|  |  | **PHQ-9 Severity OR (95% CI)** | **GAD-7 Severity OR (95% CI)** |
| --- | --- | --- | --- |
| Time x Gender | Male - Aug 2019-Mar 2020 | REF | REF |
|  | Male - May-June 2020 | 1.73 (0.98, 3.06) | 1.00 (0.54, 1.85) |
|  | Female - Aug 2019-Mar 2020 | 4.10 (1.95, 8.60) | 4.01 (1.94, 8.28) |
|  | Female - May-June 2020 | 9.81 (4.63, 20.78) | 7.10 (3.45, 14.63) |
| Age | | 0.96 (0.58, 1.62) | 0.99 (0.61, 1.62) |
| Student | No | REF | REF |
|  | Yes | 1.01 (0.55, 1.84) | 0.87 (0.49, 1.54) |
| Employed | No | REF | REF |
|  | Yes | 0.70 (0.38, 1.29) | 0.98 (0.54, 1.76) |
| Live alone | No | REF | REF |
|  | Yes | 0.83 (0.19, 3.57) | 1.09 (0.28, 4.32) |
| SEIFA | Low | REF | REF |
|  | Medium | 0.53 (0.21, 1.38) | 0.45 (0.18, 1.09) |
|  | High | 0.43 (0.18, 1.01) | 0.37 (0.17, 0.83) |
| Older siblings | No | REF | REF |
|  | Yes | 1.19 (0.68, 2.06) | 1.01 (0.60, 1.70) |
| Mean peer substance use (SD) | | 0.96 (0.88, 1.05) | 0.99 (0.91, 1.08) |
| Mean peer disapproval of substance use (SD) | | 0.81 (0.66, 1.00) | 0.85 (0.70, 1.04) |

**Figure E2** Change in meeting cut-off score for depression and GAD during COVID-19 restrictions compared to APSALS Wave 10 – time by gender interaction

| (a) Likely depressive disorder (PHQ-9) | (b) Likely GAD (GAD-7) |
| --- | --- |
|  |  |

Note: ORs shown on a log scale. Models are adjusted for covariates. Full results are included in Supplementary Table E8.

**Table E9** Predictors of meeting cut-off score for depression and GAD during COVID-19 restrictions – time by gender interaction

|  |  | **Likely presence of depressive disorder (PHQ-9 >= 10) OR (95% CI)** | **Likely presence of Generalised Anxiety Disorder (GAD-7 >= 10) OR (95% CI)** |
| --- | --- | --- | --- |
| Time x Gender | Male - Aug 2019-Mar 2020 | REF | REF |
|  | Male - May-June 2020 | 1.91 (0.75, 4.86) | 1.22 (0.47, 3.18) |
|  | Female - Aug 2019-Mar 2020 | 5.53 (1.95, 15.71) | 5.64 (2.15, 14.83) |
|  | Female - May-June 2020 | 15.87 (5.36, 46.93) | 7.15 (2.76, 18.52) |
| Age | | 1.06 (0.58, 1.93) | 0.94 (0.54, 1.61) |
| Student | No | REF | REF |
|  | Yes | 0.90 (0.44, 1.81) | 0.89 (0.47, 1.69) |
| Employed | No | REF | REF |
|  | Yes | 0.66 (0.32, 1.35) | 0.86 (0.45, 1.66) |
| Live alone | No | REF | REF |
|  | Yes | 0.31 (0.04, 2.17) | 0.84 (0.17, 4.09) |
| SEIFA | Low | REF | REF |
|  | Medium | 0.74 (0.24, 2.21) | 0.59 (0.23, 1.53) |
|  | High | 0.65 (0.24, 1.74) | 0.40 (0.17, 0.95) |
| Older siblings | No | REF | REF |
|  | Yes | 1.31 (0.69, 2.50) | 1.38 (0.77, 2.47) |
| Mean peer substance use (SD) | | 0.99 (0.90, 1.10) | 0.98 (0.90, 1.08) |
| Mean peer disapproval of substance use (SD) | | 0.85 (0.67, 1.08) | 0.82 (0.66, 1.02) |

**Table E10** Predictors of change in seeking help for mental health during COVID-19 restrictions – time by gender interaction

|  |  | **Sought Help for Mental Health OR (95% CI)** |
| --- | --- | --- |
| Time x Gender | Male - Feb 2020 | REF |
|  | Male - May-June 2020 | 0.43 (0.10, 1.85) |
|  | Female - Feb 2020 | 2.15 (0.60, 7.67) |
|  | Female - May-June 2020 | 1.17 (0.28, 4.85) |
| Age | | 1.02 (0.37, 2.83) |
| Student | No | REF |
|  | Yes | 1.13 (0.35, 3.68) |
| Employed | No | REF |
|  | Yes | 1.19 (0.34, 4.10) |
| Live alone | No | REF |
|  | Yes | 1.48 (0.09, 23.55) |
| SEIFA | Low | REF |
|  | Medium | 0.56 (0.09, 3.33) |
|  | High | 0.35 (0.07, 1.75) |
| Older siblings | No | REF |
|  | Yes | 1.43 (0.49, 4.18) |
| Mean peer substance use (SD) | | 0.91 (0.77, 1.07) |
| Mean peer disapproval of substance use (SD) | | 0.67 (0.43, 1.04) |
